
JOURNAL INFORMATION
==============================
NLM Title Abbreviation: ScientificWorldJournal
Journal ID: ScientificWorldJournal
NLM Journal ID: 101131163
Journal ID: TSWJ

The Scientific World Journal

ISSN: 2356-6140
EISSN: 1537-744X
Publisher: Wiley

ARTICLE INFORMATION
==============================
PMCID: PMC6083953
PMID: 30147516
DOI: 10.1100/tsw.2001.188
Article ID: 361059
Article version: 1
Subjects: Short Report

Analysis of Mitochondrial Function in Antigen Specific na ve, Effector and MEmory CD8 + T Cells

Grayson Jason M.
Lanier J. Gibson
Harrington Laurie E.
Altman John D.
Ahmed Rafi *
Emory Vaccine Center, Emory University School of Medicine, Atlanta, GA 30022, USA
*Rafi Ahmed: ra@microbio.emory.edu
Publication date: 2001
Electronic publication date: 2001 Nov 2
Volume: 1
First page: 53
Last page: 53
Copyright: Copyright © 2001 Jason M. Grayson et al.
Copyright year: 2001
License: This is an open access article distributed under the Creative Commons Attribution License, which permits unrestricted use, distribution, and reproduction in any medium, provided the original work is properly cited.
License URL: https://creativecommons.org/licenses/by/3.0/

==============================
Miami *Nature Biotechnology* Short Reports
*TheScientificWorld* (2001) 1 (S3), 53SR
ISSN 1532-2246; DOI 10.1100/tsw.2001.188

### ANALYSIS OF MITOCHONDRIAL FUNCTION IN ANTIGEN SPECIFIC NAÏVE, EFFECTOR AND MEMORY CD8$^+$ T CELLS

Jason M. Grayson, J. Gibson Lanier, Laurie E. Harrington, John D. Altman and Rafi Ahmed*

Emory Vaccine Center, Emory University School of Medicine, Atlanta, GA 30022
* ra@microbio.emory.edu

INTRODUCTION. CD8$^+$ T cells play a critical role in the clearance of intracellular pathogens such as viruses and certain bacteria. Before encounter of antigen these cells are naïve. After antigen encounter they become activated, begin to proliferate and acquire effector functions including cytokine production and cytolysis. These cells are effector cells. After clearance of the pathogen a death phase ensues. During this time 90-95% of the antigen specific effector cells will undergo apoptosis. The surviving cells are memory cells which can persist at constant numbers for the life of the mouse (1) (2). These cells will rapidly respond to a secondary infection and provide the basis for vaccination.

The mechanisms that control the contraction of the CD8 response remain poorly understood. Because loss of mitochondrial potential is a hallmark of cells preparing to undergo apoptosis we examined mitochondrial potential in antigen specific cells as they passed through all phases of a CD8 response.

**METHODS.** LCMV infection of mice has been described previously (3). To determine mitochondrial potential splenocytes were incubated in 40 nM 3,3'-dihexyloxacarbocyanine iodide (DiOC6(3)) for 30 minutes. After incubation with (DiOC6(3)) cells were washed once and stained with anti-CD8α-PE and $L^d$NP118-126-APC tetramer for 30 minutes on ice. The samples were washed three times and acquired on a FACSCalibur instrument.

**RESULTS.** Figure 1 shows that naïve CD8$^+$ T cells contained a basal mitochondrial potential with a mean fluorescent intensity of 230. After infection, the mitochondrial potential is increased in early effector T cells on day 5 post-infection, but by day 8 the potential in late effectors has returned to a level comparable to naïve cells.

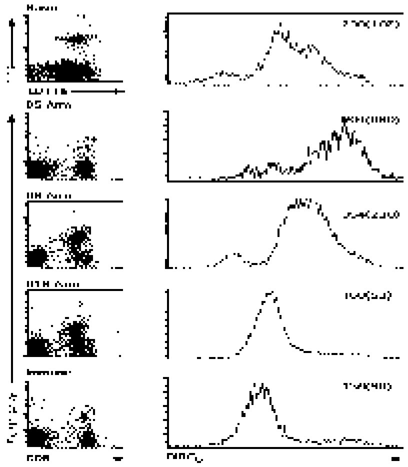

Antigen specific cells in the death phase (day 13) contained a decreased potential relative to naïve cells. The potential in memory cells was indistinguishable from that of antigen specific cells during the death phase.

**DISCUSSION.** In this short report we present analysis of mitochondrial function in antigen specific cells as they progress from naïve to effector to memory cells. We confirm previous studies that T cells undergoing apoptosis contain a lower mitochondrial potential (4,5) and extend these findings to show that the potential in memory cells does not return to the level found in naïve cells. These findings suggest that memory cells contain some factor that allows them to survive despite a decreased membrane potential.

**SUPPORT.** This research was supported by National Institutes of Health Grants AI30048 (to R.A.) and NS 21496 (to R.A.). J.M.G. was supported by National Research Service Award 1F32AI0249-01A1.

**REFERENCES.**

1. Murali-Krishna, K. et al. (1998) Immunity 177-188
2. Murali-Krishna, K. et al. (1999) Science 286, 1377-1381
3. Grayson, J.M., Zajac, A.J., Altman, J.D., and Ahmed, R. (2000) J. Immunol. 164, 3950-3954
4. Zamzami, N. et al. (1995) J. Exp. Med. 182, 367-377
5. Zamzami, N. et al. (1995) J. Exp. Med. 181, 1661-1672
